# PReS-FINAL-2019: Tuberculosis infection observed in patients receiving biological DMARDs for treatment of juvenile idiopathic arthritis

**DOI:** 10.1186/1546-0096-11-S2-P32

**Published:** 2013-12-05

**Authors:** A Sarychev, L Shovkun

**Affiliations:** 1 Pediatrics, Russian Federation; 2 Rostov State Medical University, ROSTOV-NA-DONU, Russian Federation

## Introduction

The use of immunosuppressant drugs and also biological DMARDs in the treatment of juvenile idiopathic arthritis (JIA) may weaken patient's immunity and raise the risk of serious infections, including tuberculosis (TB).

## Objectives

To assess the incidence rate of different forms of TB infection in patients receiving biological treatment of JIA.

## Methods

Thirty four children, aged from 3 to 17 years, were observed prospectively receiving biological treatment for JIA. Mean disease duration was 5,29 (± 4,24) yrs. Initial screening for TB included PPD test and chest CT.

## Results

Fourteen of 34 patients (41,2%) received toziliaumab (TOZ), 6 (17,6%) - abatacept (ABA), 4 (11,8%) - etanercept (ETN), 5 (14,7%) - adalimumab (ADA) and 5 (14,7%) - infliximab (IFL). Mean treatment duration with biologics was 16,3 (± 10,47), from 3 to 46 months. Different types of TB infection were diagnosed in 6 (17,6%) patients while receiving biological DMARDs. Three of them received treatment for systemic JIA, 1 - for polyarticular JIA, 1 - juvenile ankylosing spondylitis and 1- JIA with concomitant uveitis. Different clinical types of TB infection were observed in these 6 patients: one patient receiving TOZ developed infiltrative pulmonary TB with dissemination, two patients (1 on TOZ and 1 on ADA) had primary TB complex and three (on IFL, ADA, ETN) were diagnosed with latent TB infection. The common feature TB infection in all patients receiving biologics was the mildness of clinical manifestation of the disease.

In addition 3 cases of primary TB complex were observed in patients with systemic JIA before initiation of biological DMARD and required prior specific TB therapy.

## Conclusion

Children with systemic JIA may be considered to have the higher risk of different types of TB infection. Treatment with biological DMARDs (especially with TNF-α and IL-6 inhibitors) also may contribute to TB infection. Regular TB screening (at least once per 6 months) is required to the scarcity of clinical manifestation of the infection.

## Disclosure of interest

None declared.

